# Mediation and moderation analyses: exploring the complex pathways between hope and quality of life among patients with schizophrenia

**DOI:** 10.1186/s12888-020-2436-5

**Published:** 2020-01-15

**Authors:** Wei-Liang Wang, Yu-Qiu Zhou, Nan-Nan Chai, Guo-Hua Li, Dong-Wei Liu

**Affiliations:** 1 School of Nursing, Harbin Medical University (Daqing), Daqing, Heilongjiang China; 2 School of Nursing, Chifeng University, Chifeng, the Autonomous Region China; 3 Chifeng Anding Hospital, Chifeng, the Autonomous Region China

**Keywords:** Schizophrenia, Quality of life, Moderated mediation, Hope, Resilience, Depression

## Abstract

**Background:**

The underlying mechanism between hope and quality of life is as yet unknown. We aim to examine the potential mediating effect of depression and resilience and the moderated effect of sex in this well-established association.

**Methods:**

Two hundred seven patients diagnosed with schizophrenia were administered a questionnaire battery that measured hope, depression, resilience and QOL. A multiple mediation model was used to examine the mediating effect of resilience and depression on the association between hope and QOL. A subgroup analysis was performed and a moderated mediation model was examined to find and test the moderated effect of sex on the mediation model. We used Mplus to perform moderation and mediation analyses so that the mediators and moderator could function together in the same model.

**Result:**

Sex was the moderator on the direct path between hope and QOL. The relationship between hope and QOL was mediated by resilience and depression in both sexes. When compared with female patients, the effect of hope on QOL was completely mediated by resilience and depression in males. In female patients, the model was partially mediated, and the direct effect of hope on QOL was significantly negatively correlated with the level of hope.

**Conclusion:**

We present a conceptual model containing the mediated effects of resilience and depression and the moderated effect of sex between hope and QOL, which we believe facilitates the understanding of these associations. This model should be useful in the formulation of strategies to improve QOL.

## Background

Abundant evidence has shown that people with mental illness have the possibility of living a qualified and satisfying life with residual symptoms [1–3]. QOL is a critical clinical outcome, closely related to patient function and disability, and is often a direct evaluation indicator of personal recovery outcomes among patients with schizophrenia [4, 5]. Determinants of QOL in individuals with schizophrenia have been reported in previous studies [6, 7]. Among these influencing factors, psychosocial components are more preventable in clinical practice. Hope as a critical psychological characteristic has been regarded as a central foundation of rehabilitation and is related to almost all outcomes. Hope in patients with schizophrenia is defined as positive expectations for the future, confidence in life and the future, and meaning in life based on a patient’s perspective [8]. The level of hope has been shown to be highly correlated with QOL and to improve the QOL of people with schizophrenia [7, 9]. Even in patients with high disability, increasing hope by setting meaningful and attainable goals often leads to a greater QOL [10]. However, limited studies have focused on the potential underlying mechanisms between hope and QOL in patients with schizophrenia.

Depression, is very common after the recovery of clinical insight, with a post-schizophrenic depression rate of 27% [11], which can be caused by multiple factors, such as antipsychotics or their side effects. DeRosse et al. found that depressive symptoms were significantly predictive of QOL as well as cognition in patients with schizophrenia [12]. Even after controlling for negative symptoms in multiple regression analysis, depressive symptoms were also an important independent predictor of all QOL domains [13]. As one of the most prevalent domains of depressive syndrome in patients with schizophrenia, hopelessness is regarded as the most powerful psychological predictors of depression [10, 14]. The association between hope and depression has also been shown in a previous study [15].

The nature of QOL in patients suffering from schizophrenia is complex. Wartelsteiner [7] showed that QOL correlated moderately with resilience and hopelessness and weakly with symptoms, and significant efforts are necessary to enhance resilience and to diminish hopelessness as well as affective and positive symptoms in patients with schizophrenia for better QOL. Resilience is the ability to adapt to stress and adversity and to maintain or restore mental health and is rooted in the rehabilitation process and reflects a kind of ‘fighting attitude’ [16]. Resilience has received increasing attention in schizophrenia in recent years since there is evidence that it has a positive impact on the long-term outcome of patients, similar to QOL [3, 17]. Resilience is a positive personality trait that enhances an individual’s adaption or capacity for recovery and may help patients with schizophrenia cope with and gain insight into their illness. Furthermore, resilience, as a dynamic structure to maintain the balance of an individual’s state, should always be treated as an important target for intervention or the core of intervention programmes, as it is critical to the recovery of patients [18]. As an important predictor of QOL, even among remitted patients, the predictive effect of resilience on QOL is still significant [19]. Hope and resilience, as two major areas of consumer-based recovery, are significantly correlated in patients with schizophrenia [20]. These correlated relationships between hope and resilience have also been explored in other studies and shown to play an important role in QOL improvement [7, 21, 22].

Many studies have shown that QOL in schizophrenia patients differs between men and women [23–25], and the predictors of QOL between different sexes are also different [26]. Schizophrenia research suggests that females appear to have a better illness course than males, as they present better remission and lower relapse rates, a lower risk of being admitted to the hospital than males, fewer negative symptoms, and better functions [27, 28]. Compared to men, females have superior mentalizing abilities [29]. Thus, we assumed that there were conditional relationships between hope, resilience, depression and QOL in different sex groups.

Little research has been conducted to identify mechanisms of the relationship between hope and QOL in patients with schizophrenia. Identifying specific mechanisms for the association between hope and QOL may provide a theoretical model to help explain the relationships between these variables. The model can help us understand how these structures are related, which is vital in developing targeted interventions to address QOL deficits [30].

The aim of this study was to test and verify the mediation of resilience and depression and to analyse the moderating effect of sex on the association between hope and QOL in patients with schizophrenia. According to the evidence reviewed above, we proposed the following hypotheses: H1: Resilience has a mediating effect between hope and QOL; H2: Depression has a mediating effect between hope and QOL; H3: Sex has a moderating effect on one or more paths among these variables.

## Patients and methods

### Subjects

All participants were recruited from Chifeng Anding Hospital located in the Inner Mongolia autonomous region in China. The inclusion criteria were (1) ICD-10 diagnosis of schizophrenia; (2) clinical stability and absence of aggressive or hostile behaviour; (3) age 18 years or older; (4) ability to understand the survey instructions and willingness to provide written informed consent; and (5) sufficient cognitive capacity (a score of PANSS-G12 less than 4 [31]). The exclusion criteria were as follows: coexisting mental retardation, dementia, or other severe organic disorders, or drug or alcohol abuse.

Clinical stability was defined as an increase in drug dosage not more than 50% in the 3 months before assessment [32].

### Tools

#### Quality of life

QOL was measured using the Schizophrenia Quality of Life Scale (SQLS) [33], a customer-based instrument for schizophrenia to assess QOL. It includes 30 items in three domains: psychosocial, motivation and energy and symptom and side effects. The SQLS is a 5-point Likert-type scale where 1 = *never* to 5 = *always*, with higher scores indicating worse QOL (four items (12, 13, 15 and 20) are reverse coded). The internal consistency of the scale in the current study was 0.83.

#### Hope

Hope was measured using the total score on the Schizophrenia Hope Scale (SHS-9) [8], a 9-item questionnaire assessing optimism and hope for the future, rated on 3-point items (disagree, agree, strongly agree). The internal consistency alpha coefficient was 0.92, with a score range of 0–18. Higher scores indicated higher levels of hope.

#### Depression

The 9-item Calgary Depression Scale for Schizophrenia (CDSS) [34] was used to assess depressive symptoms. The CDSS contains two components: depression experience and self-evaluation. Scores range from zero (absent) to three (severe) and were assessed during a structured interview and summed. High scores indicated high levels of depression, and Cronbach’s alpha was 0.86 in the current study.

#### Resilience

Resilience was assessed by the Connor-Davidson Resilience Scale (CD-RISC) [35, 36], which views resilience as a personal quality and evaluates it in relation with internal resources. It consists of 25 items, each rated on a 5-point scale (0 = not true at all to 4 = true nearly all the time). The CD-RISC contains three factors: Tenacity, Strength, and Optimism. Total scores range between 0 and 100, with higher scores reflecting greater resilience, and Cronbach’s alpha was 0.80 in the current study.

### Procedure

The diagnosis of patients was based on their medical records and verified by an experienced psychiatric clinician (LGH); other criteria for inclusion were determined by the clinician (LGH) and the investigator (WWL). Patients who met the inclusion criteria were evaluated with the SQLS, SHS-9, CDSS and CD-RISC by the same researcher (WWL) in a quiet and comfortable room after obtained the informed consent from patients.

### Statistical analysis

**Fig. 1:**
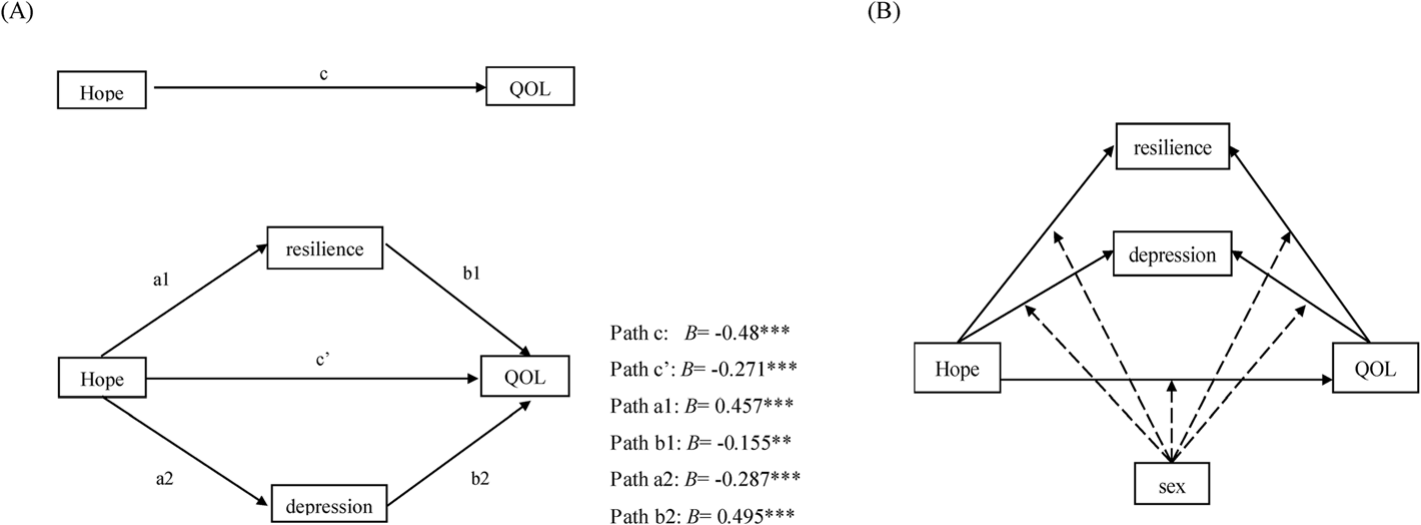
(Panel **a**: H1 and H2) Conceptual framework of the potential mediating effect of resilience and depression on the relationship between hope and QOL. (Panel **b**: H3) Model of the potential moderated effects on the paths

In our conceptual model (Fig. 1a), the observed effect of hope on the QOL is called the total effect (path c). The total effects comprised a direct effect pathway (path c’) of hope on QOL and a total indirect pathway (mediated: path a1b1 + path a2b2) of hope on QOL through resilience and depression. Figure 1b shows the models of the potential moderated relationship of sex on the multiple mediators model.

#### Mediating effect

Mediation analysis was performed to study whether the relationship between hope and QOL was mediated by depression and resilience. To avoid biased parameter estimates caused by simple mediation, a multiple mediators model was established to verify the hypotheses [37]. This model was used to test whether there was a significant indirect effect between hope and QOL. When the mediator variables are introduced, the direct effect (path c’) is reduced but still significant, which is called partial mediation. Complete mediation indicates that the direct effect (path c’) is not significant after introducing the mediator variables.

#### Moderating effect

Subgroup analysis was performed to test the moderating effect among simple paths in the multiple-mediation model. The difference in the path coefficient with statistical significance between the two groups was regarded as evidence that the moderation effect in this path existed [38].

#### Moderated mediation

According to Edwards’ suggestion, tests of mediation effects in each subgroup will lead to a biased estimated parameter and low statistical power. Subgrouping analysis was only used to test which path the moderator affected, and the estimated parameters, including the total, indirect and direct effects of the moderated mediation model, were conducted by integrating moderation and mediation methods [37, 39]. Simple slope analyses were used to aid interpretation of the interaction plots.

Participants’ demographic and clinical characteristics were characterized by descriptive statistics using Stata version 14.2. These simple path analysis models were estimated using maximum likelihood estimation and bootstrapped standard errors using Mplus v8.0 software. All statistical tests were two-tailed, and significance was determined at the .05 level. A bootstrapping procedure was used to test the significance of the total and indirect effects and the differences in these effects across levels of the moderator variables with 5000 bootstrap samples [39]. The 95% confidence intervals for the coefficients calculated by bootstrapping methods were considered statistically significant if the confidence intervals did not include zero. All of the Mplus codes for mediation, moderation, and moderated mediation models were from the website: http://www.offbeat.group.shef.ac.uk/FIO/mplusmedmod.htm#modindex [40].

## Results

### Participant characteristics

Two hundred seven patients were recruited from July 2017 to May 2018. Participant characteristics are presented as counts and percentages for categorical variables and the mean ± SD for continuous variables. More than half of the patients were male and middle aged. To ensure the credibility of the collected data, we defined the patients’ insights in the admission criteria. More than one-half were considered as having complete insight, and others were considered as having partial insight (mild insight impairment). Overall, the patients were predominantly single (59.9%, never married and divorced) and with less than a high school education (71.0%) (Table 1).

**Table 1:** Participant Sociodemographic and Clinical Characteristics

Characteristic	Total *N* = 207	Male *N* = 137	Female *N* = 70	*P* value
Age in years, *M* (SD)	42.26 (10.02)	42.45 (10.06)	41.86 (9.99)	0.683
Marriage status, %(N)				
Alone	26.6 (55)	20.44 (28)	38.57 (27)	<0.001
Divorced	33.3 (69)	27.74 (38)	44.29 (31)	
Marriage	40.1 (83)	51.82 (71)	17.14 (12)	
Education level, % (N)				
Less than high school	71.0 (147)	74.45 (102)	64.29 (45)	0.127
High school and above	29.0 (60)	25.55 (35)	35.71 (25)	
Insight (G12), % (N)				
Complete (G12 = 1)	58.5 (121)	55.47 (76)	64.29 (45)	0.224
Partial (G12 ≤ 3)	41.5 (86)	44.53 (61)	35.71 (25)	
SHS-9, *M* (SD)	10.14 (4.72)	9.71 (4.79)	10.99 (4.49)	0.065
CD-RISC, *M* (SD)	40.58 (14.29)	41.42 (14.61)	38.94 (13.59)	0.238
CDSS, *M* (SD)	3.25 (3.26)	3.22 (3.50)	3.31 (2.75)	0.843
SQLS, *M* (SD)	62.02 (40.39)	57.48 (37.56)	70.91 (44.39)	0.037

Analysis between male vs female was performed with t-tests for continuous variables (normal distribution) and χ2 test for categorical variables

*SD* standard deviations, *M* means, *N* numbers, *G12* 12th item of general psychopathology symptoms of PANSS, *SHS-9* schizophrenia hope scale, *CD-RISC* Connor-Davidson Resilience Scale, *CDSS* Calgary Depression Scale for Schizophrenia, *SQLS* Schizophrenia Quality of Life Scale

### Mediation models

The results showed that all of the simple path coefficients (path a1, b1, a2, b2 and c’) were statistically significant, with *p* < 0.05 (Fig. 1). The results from 5000 bootstrapping samples indicated that all of the indirect effects were statistically significant, with the bootstrapping 95% CI not including zero. The total effect of hope on QOL was − 0.485 (*p* < 0.001), and the proportion of the total indirect effect of QOL on hope estimated by resilience and depression was 43.91%.

### Moderation analysis

The sex-stratified analysis showed that the direct effect on females was obviously greater than that on men, and only the difference in the direct effect was statistically significant with p=0.033, which indicated that sex as a moderator had a moderating effect on the direct path (path c’) (Table 2).

**Table 2:** Analysis of simple effects

Moderator variable	Effect						
	a1	b1	a2	b2	c’	a1b1	a2b2
Gender							
Male	0.435***	− 0.138*	−0.25**	0.564***	−0.25**	− 0.06	− 0.141**
Female	0.562***	−0.075	− 0.405**	0.335***	− 0.451***	− 0.042	−0.144**
Difference	0.973	0.078	0.526	0.025	4.548*	0.005	0.253

Differences in simple effects were computed by subtracting the effects for women from the effects for men

Tests of differences for the indirect effect were based on bias-corrected confidence intervals derived from bootstrap estimates

*p < 0.05; **p < 0.01; ***p < 0.001

### Moderated mediation analysis

**Fig. 2:**
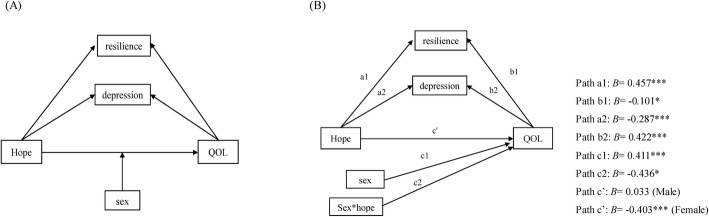
The conceptual (panel **a**) and statistical (panel **b**) forms of the conditional process model (moderated mediation). In this model the indirect effect of hope on QOL through depression and resilience, and the direct effect of hope on QOL is supposed to be moderated by sex

Figure 2 shows the conceptual (2A) and statistical (2B) forms of the moderated mediation model. The results of the moderated mediation analysis showed that the moderating effect of sex (path c1) and the interaction effect of sex and hope (path c2) on QOL were statistically significant, with *p* < 0.05 (Fig. 2). Both indirect effects (a1b1 and a2b2) were statistically significant, and there was a significant difference between both indirect paths, with the BC 95% bootstrap CI not including zero. The coefficient of the indirect effect of QOL via resilience on hope (a1b1) was − 0.046 and accounted for 38% of the total indirect effect. The indirect effect of QOL via depression on hope (a2b2) was − 0.121 and accounted for 62% of the total indirect effect (Table 3). In the male patient group, the direct effect was not statistically significant, and the model was complete as mediated by depression and resilience. In female patients, the model was partially mediated with a significant direct effect (path c’).

**Table 3:** Bootstrap mediation and moderated mediation effect

		Point Estimate	Product of confidents		BOOSTRAP 5000 TIMES 95% CI			
					bias corrected		percentile	
			S.E.	Est./S.E.	Lower	Upper	Lower	Upper
Mediated model	Indirect effect							
	QOL – Resilience - Hope	−0.071***	0.027	−2.659	−0.130	− 0.026	−0.126	− 0.022
	QOL – Depression - Hope	− 0.142***	0.034	−4.187	− 0.212	−0.078	− 0.208	−0.073
	Total indirect effect							
	Total indirect effect	−0.213**	0.043	−4.929	−0.303	−0.134	− 0.295	−0.127
Moderated mediation model	Indirect effect							
	QOL – resilience - hope	−0.046*	0.023	−1.973	−0.104	−0.009	− 0.096	−0.004
	QOL–depression - hope	−0.121***	0.033	−3.686	−0.195	−0.064	− 0.188	−0.058
	Total indirect effect							
	Total indirect effect	0.167***	0.043	−3.875	−0.265	− 0.094	−0.265	− 0.086
	Contrasts							
	Resilience vs depression	0.730*	0.356	2.050	0.057	1.466	0.033	1.438

Percentile 95% CIs for bootstrap distributions are defined using the values that mark the upper and lower 2.5% of each distribution

There were differences between the percentile and bias corrected methods, depending on the size of the paths in the model, with the percentile method generally showing a slight superiority in conditions in which the bias corrected method was slightly liberal

*CI* confidence interval, *QOL* quality of life, *SE* standard error

*p<0.05; **p <0.01; ***p<0.001

**Fig. 3:**
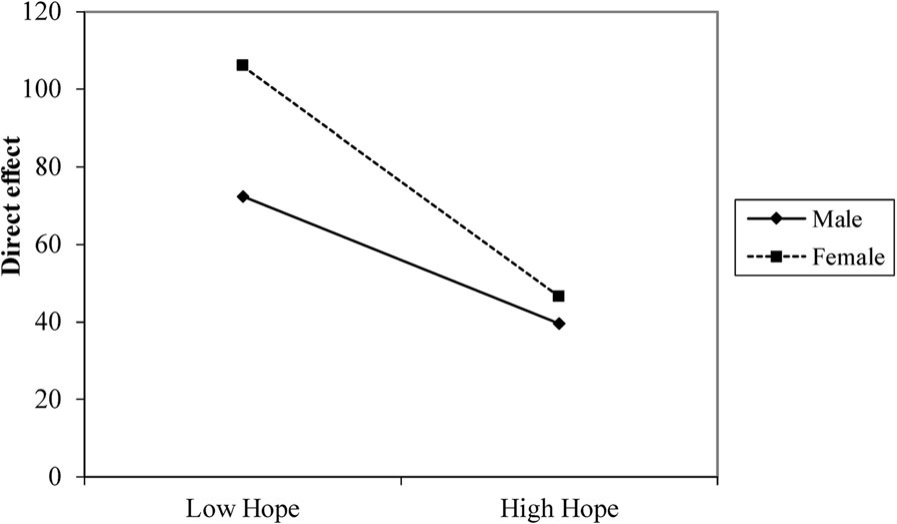
Moderating effect of sex on the direct effect between hope and QOL

A visual presentation of the interaction demonstrating the pattern of effect in males and females is presented in Fig. 3. The figure plots the relationship between hope and predicted QOL for males and females and shows that the line for females is steeper, suggesting that the level of hope for females has a strong effect on QOL.

## Discussion

Over the last several decades, because of the close association between QOL and functional disability, QOL has become an important indicator for the evaluation of treatment outcomes in individuals with schizophrenia. One of the major purposes of this study was to clarify the mechanism of the relationship between hope and QOL by concurrently examining the mediational roles of depression and resilience. The results showed that the level of hope was closely correlated with QOL, and consistent with expectations, the relationship between hope and QOL was mediated by depression and resilience, which proved hypotheses 1 and 2.

These findings are consistent with previous studies showing associations among hope, depressive symptoms, resilience and QOL in other fields [41, 42]. To the best of our knowledge, this study is the first to show such a relationship between hope and QOL in patients with schizophrenia. As hypothesized, hope was indirectly related to QOL through resilience and depression, such that a higher level of hope was associated with lower depression and higher resilience, which in turn were associated with higher QOL.

Snyder [43] suggested that hope was a natural balancing force against depression and that this balance was the fundamental and adaptive aspect of human existence. Previous studies have shown that people with schizophrenia have significantly less hope than the general population, and the level of hope was an important predictor of the severity of depressive symptoms, which was associated with lower levels of functioning in general and lower functioning regarding daily living, health and social life, in particular [44–46]. At the same time, in theory, higher resilience levels can preclude, reverse or slow the progression of psychiatric disorders [47]. Resilience is the ability to positively adapt despite adversity, which will buffer the impacts of stress and symptoms on health outcomes [48]. According to Snyder [49], hope is about establishing pathways to goals and the perceived capacity to use one’s pathways to reach desired goals. A strong sense of purpose can improve resilient outcomes, and regaining a sense of purpose may be crucial to the recovery process [50]. The indirect effects of depression and resilience suggest that patients with schizophrenia who are at a low level of hope may experience lower QOL; a high level of hope may then augment or repair the impaired resilience of schizophrenia patients and can function as a protective effect to better the QOL. In another mediated path, a high level of hope may release depressive symptoms and further improve QOL. Certainly, this causal relationship among these variables needs to be verified by specially designed studies in the future.

The high proportion of the total indirect effect indicated that resilience and depressive symptoms play important roles in the improvement of QOL from hope in patients with schizophrenia. Furthermore, the effect of hope on QOL via depression was significantly greater than the effect of hope on QOL via resilience, which was consistent with the high rate of depression and its serious impact on QOL in patients with schizophrenia [13, 45].

Another finding of this study was that sex had a moderating effect on the direct path. In male patients, the effect of hope on QOL was completely mediated by depression and resilience. However, in the female group, the direct effect decreased with increasing hope. When the level of hope is high in female patients, the mediated model tends to be completely mediated by depression and resilience, and when the level of hope is low, the effect of hope on QOL is obvious, and the relationship between hope and QOL is partially mediated by depression and resilience. Hope in patients with schizophrenia contains two different dimensions: “future expectations” and “motivational hope.” A previous study showed that personality (such as neuroticism) and cognition (such as verbal memory and isolation) were each uniquely related to expectations of the future and motivational hope [40]. Personality and cognition were considered important factors that influenced QOL; thus, significant differences in personality traits, neurocognition and social cognition between male and female schizophrenia patients may contribute to the moderating effect of sex on the direct path [27, 28, 51, 52]. Similar results had been found in the general population. Sex was found to be directly related to hope in females [53]. Indeed, sex might be the potential moderator of the relationship between hope and well-being. Compared to their male counterparts, females exhibited significantly less hope, which negatively impacted their adjustment to adversity and their ability to effectively cope with challenges in achieving goals, which could be detrimental to their well-being; furthermore, they might experience failures in researching goals and be less able to find alternative ways to achieve them [54]. The relevance of sex differences in schizophrenia had meaning beyond academic interest since these differences can affect treatment. The observed complex associations among investigated predictors, mediators and moderators strongly suggested that integrated and personalized programmes should be provided as standard treatment to people with schizophrenia.

A theoretical model was built based on the evidence that depression and resilience as significant mediators and the conditional direct effect of hope on QOL could better explain the relationship between clinical variables and QOL in patients with schizophrenia. As Wilson and Cleary noted [30], identifying causal pathways that link different types of outcomes to each other contributed to the optimal design of interventions to improve patient outcomes. Only by understanding the underlying correlated mechanisms among the determinants of QOL can we hope to develop rational and effective strategies to improve QOL. Our findings showed that the balance among hope, resilience and depression was crucial for better QOL in patients with schizophrenia. The mediating effect of resilience and depression and the moderating effect of sex may help to reveal the mechanisms of the relationship between hope and QOL to some degree. We present a conceptual model that we believe facilitates the understanding of these associations. This model should be useful in the formulation of strategies to improve QOL.

The participants of this study were from clinically stable inpatients, which required careful interpretation of current findings. As Spencer et al. [55] had mentioned, the hospitalized patients such as schizophrenia and related psychoses should not be regarded as exclusive areas for research, which was particularly applicable in China. The clinically stable inpatients, who occupied a large proportion of the schizophrenia group in China, was very representative and had not received enough attention. Clinical stability was an important period for clinical intervention when the existing community management model was immature in China. Evidence had shown that the treatment experience during hospitalization was closely related to the patient’s treatment attitude, help-seeking behaviour and treatment continuity [56–58]. Therefore, the complex pathways between the hope level and QOL revealed by this research provides thoughts on targeted interventions to optimize the treatment experience and promote rehabilitation. And because the sample was from clinically stable inpatients, we could completely control the effect of medication compliance on the outcomes, which was of great significance for exploring the true path relationship among variables.

### Limitations

There are several limitations to our study. First, the participants in our study were limited to one location, and it is unclear whether these findings will generalize beyond this subsample of the population. Second, the study sample was relatively small, especially for females (only 70), which may have limited the generalizability of our findings. Third, as we noted before, the nature of QOL is complex, and there may be more complicated relationships among the clinical variables that we missed. Future studies should focus on the mechanisms leading to health-related outcomes. Fourth, we did not consider the impact of diagnostic subtypes of schizophrenia, which may be a source of some bias. Fifth, the criteria for clinical stability used in our study are not comprehensive enough, which may cause potential sample bias. Finally, the cross-sectional study design limits the interpretation and generalization of our results. The one-sided causality that was evaluated should be considered when interpreting our results. Future research should conduct a longitudinal study to verify the current conclusions and should control for other variables that may influence the results.

## Conclusion

Despite these limitations, this study shows the persistence of the impact of hope on QOL in male patients with schizophrenia, in whom it is completely determined by resilience and depressive symptoms; it is also partially determined by these factors in female patients. With an increase in the level of hope in female patients, the percentage of the effect determined by resilience and depressive symptoms increases. Our results help to reveal the underlying influencing mechanisms between these psychological variables and QOL. However, due to the cross-sectional study design, our results should be interpreted with caution. Confirmation of these results may help to develop targeted interventions that may contribute to improving QOL.

## Data Availability

The datasets analysed during the current study are available from the corresponding author upon reasonable request.

## Abbreviations

CD-RISC: Connor-Davidson Resilience Scale
CDSS: Calgary Depression Scale for Schizophrenia
G12: 12th item of the general psychopathology symptoms of PANSS
QOL: Quality of life
SHS-9: Schizophrenia hope scale
SQLS: Schizophrenia Quality of Life Scale
