# Correction to: Mediation and moderation analyses: exploring the complex pathways between hope and quality of life among patients with schizophrenia

**DOI:** 10.1186/s12888-021-03517-3

**Published:** 2021-11-12

**Authors:** Wei-Liang Wang, Yu-Qiu Zhou, Nan-Nan Chai, Guo-Hua Li, Dong-Wei Liu

**Affiliations:** 1 School of Nursing, Harbin Medical University (Daqing), Daqing, Heilongjiang China; 2 School of Nursing, Chifeng University Chifeng, Harbin, The Autonomous Region China; 3 Chifeng Anding Hospital Chifeng, Chifeng, The Autonomous Region China

**Correction to: BMC Psychiatry. 20, 22 (2020)**

**http://orcid.org/0.1186/s12888-020-2436-5**

Following the publication of the original article [1], the authors identified errors in Figs. 1 and 2 and Table 2. The correct figures and table are given below.

**Fig. 1:**
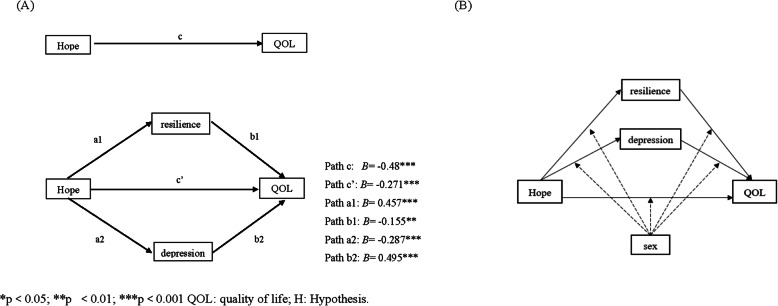
(Panel **A**: H1 and H2) Conceptual framework of the Potential Mediating Effect of resilience and depression on the Relationship between hope and QOL. (Panel **B**: H3) Model of the potential moderating effects on the paths. *p<0.05; **p<0.01; ***p<0.001 QOL: quality of life; H: hypothesis

**Fig. 2:**
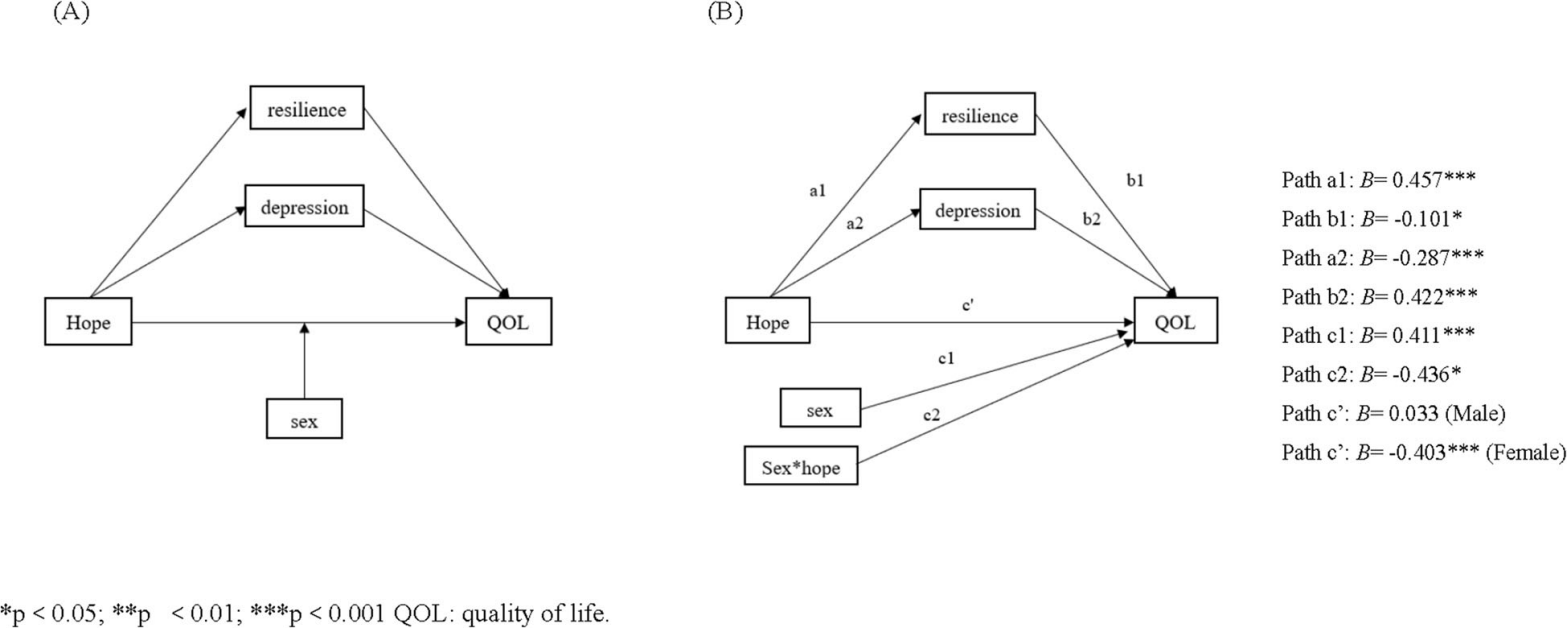
The conceptual (panel **A**) and statistical (panel **B**) forms of the conditional process model (moderated mediation). *p<0.05; **p<0.01; ***p<0.001 QOL: quality of life. In this model, the indirect effect of hope on QOL through depression and resilience and the direct effect of hope on QOL are supposed to be moderated by sex

**Table 2:** Analysis of Simple Effects

Moderator variable	Effect						
	a1	b1	a2	b2	c’	a1b1	a2b2
Gender							
Male	0.435***	−0.138*	− 0.25**	0.564***	− 0.25**	− 0.06	− 0.141**
Female	0.562***	− 0.075	−0.405**	0.335***	−0.451***	− 0.042	−0.144**
Difference	0.973	0.078	0.526	0.025	4.548*	0.005	0.253

*p<0.05; **p <0.01; ***p<0.001

Differences in simple effects were computed by subtracting the effects for women from the effects for men

Tests of differences for the indirect effect were based on bias-corrected confidence intervals derived from bootstrap estimates

The original article [1] has been corrected.
